# Reduction of proteolysis of high protein silage from Moringa and Indigofera leaves by addition of tannin extract

**DOI:** 10.14202/vetworld.2019.211-217

**Published:** 2019-02-09

**Authors:** Anuraga Jayanegara, Aldi Yaman, Lilis Khotijah

**Affiliations:** Department of Nutrition and Feed Technology, Faculty of Animal Science, Bogor Agricultural University, Bogor 16680, Indonesia

**Keywords:** deamination, feed fermentation, polyphenol, protein degradation

## Abstract

**Aim:**

The objective of this experiment was to evaluate the effect of the addition of tannin extract to Moringa and Indigofera leaf silages on their chemical composition, silage quality characteristics, and *in vitro* rumen fermentation parameters and digestibility.

**Materials and Methods:**

Moringa and Indigofera leaves were cut (3 cm length) and added with either 0, 2, or 4% chestnut tannin in three replicates. The leaves were then inserted into lab-scale silos (1 L capacity) and kept for 30 days. Silage samples were subjected to silage quality determination, chemical composition analysis, and *in vitro* rumen fermentation and digestibility evaluation using a gas production technique. Data obtained were subjected to the analysis of variance with a factorial statistical model in which the first factor was different silage species and the second factor was tannin addition levels.

**Results:**

Tannin addition at 4% dry matter (DM) increased neutral detergent insoluble crude protein (NDICP) and acid detergent insoluble CP (ADICP) of Indigofera silage. A similar response was observed in Moringa silage, but it required less tannin, i.e., 2% DM to increase its NDICP and ADICP. Moringa silage had lower pH than that of Indigofera silage (p<0.05), and tannin addition did not change pH of both Indigofera and Moringa silages. Higher addition level of tannin decreased total volatile fatty acid (VFA) and ammonia concentrations of both Indigofera and Moringa silages (p<0.05). A higher level of tannin addition reduced ruminal total VFA concentration, ammonia, *in vitro* DM digestibility, and *in vitro* organic matter digestibility of Indigofera and Moringa silages (p<0.05). Tannin addition also decreased ruminal methane emission of both Indigofera and Moringa silages (p<0.05).

**Conclusion:**

Tannin extract can reduce proteolysis of high protein silage from Moringa and Indigofera leaves.

## Introduction

Indigofera and Moringa leaves are known to have high protein contents and therefore have been used as protein supplements in ruminant diets [1-4]. These forages are typically used either as fresh or dried forms. Another possible way to use Indigofera and Moringa leaves is through anaerobic fermentation of the forages by the action of primarily lactic acid bacteria [5], i.e., their silage forms. Use of Indigofera and Moringa leaves in their conserved forms as silages is a way to provide sufficient protein supply continuously across all seasons in a year. However, such high protein silages have a problem with regard to massive protein degradation and amino acids deamination during the ensiling process [6,7]. Our previous study showed that Indigofera and Moringa leaf silages had high pH values and high ammonia concentrations that contributed to their low silage quality [8]. It had been recommended that a certain silage additive is necessary to prevent or to minimize such considerable proteolysis in high protein silages. Ideally, the additive provides additional advantages other than its function in silage.

Here, we attempted to use tannin extract as a silage additive. Tannin has multiple hydroxyl groups in its structure and ability to interact with other molecules particularly protein [9] and therefore is expected to protect microbial degradation of protein occurring in silage. Apart from its potential use as a silage additive, tannin has been reported to provide additional benefits when being used in the diet of ruminants. Tannin is able to protect protein degradation by rumen microbes [10] thus enhances protein bypass and metabolizable protein supply for ruminants [11], and in turn, improves animal productivity [12]. Parasitic nematodes can be controlled by tannin in the digestive tract of ruminants [13]. Further, tannin reduces enteric methane emission of ruminants through inhibition of methanogen and protozoa population [14]. Tannin inhibits biohydrogenation of polyunsaturated fatty acids in the rumen [15] and improves the deposition of these beneficial fatty acids in animal products [16,17].

This study aimed to evaluate the effect of the addition of tannin extract to Moringa and Indigofera leaf silages on their chemical composition, silage quality characteristics, and *in vitro* rumen fermentation parameters, and digestibility. It was hypothesized that tannin extract would limit proteolysis occurring in Moringa and Indigofera silages.

## Materials and Methods

### Ethical approval

The present experiment used rumen content (both solid and liquid parts) as the source of microbial inoculum. Rumen content was collected from two fistulated Ongole crossbred cattle before morning feeding, located at Biotechnology Research Center, Indonesian Institute of Sciences, Bogor, Indonesia. The cattle were maintained according to the animal welfare standard of Indonesian Institute of Sciences. Approval of the whole experiment was granted from the Faculty of Animal Science, Bogor Agricultural University, Indonesia.

### Sample collection and ensiling procedure

Moringa and Indigofera leaves were collected from Agrostology Field Laboratory of Faculty of Animal Science, Bogor Agricultural University. Approximately 9 kg fresh of each leaf species was manually cut (3 cm length) and divided into nine portions. Each portion was added with either 0, 2, or 4% chestnut tannin (dry matter [DM] basis) in three replicates. Leaves were then inserted into lab-scale silos (1 L capacity) and kept for 30 days. Each lab-scale silo is equipped with a slit that enables gas formed during ensiling to escape but prevents gas from outside to enter the silo. After 30 days, the silo was opened, and silage was divided into two portions with a similar proportion. The first portion was mixed with distilled water in a blender (silage: distilled water 1:7, w/w [18]) and the supernatant was subjected to silage quality determination. The second portion was oven-dried at 60°C for 24 h, ground by a hammer mill to pass a 1 m sieve, and used for chemical composition analysis and *in vitro* rumen fermentation evaluation.

### Chemical composition analysis

Silage samples were determined for their chemical constituents, i.e., DM, crude protein (CP), neutral detergent fiber (NDF), acid detergent fiber (ADF), neutral detergent insoluble CP (NDICP), and acid detergent insoluble CP (ADICP). DM was determined by oven-drying the sample at 105°C for 24 h, whereas CP was analyzed using a Micro Kjeldahl Apparatus to obtain N content [19]. The CP was obtained by multiplying N content with 6.25, assuming that generally, protein contains 16% N. Contents of NDF and ADF were determined according to Van Soest *et al*. [20]. Briefly, an amount of 1 g sample was put into a 600 ml beaker glass and added with 100 ml neutral detergent solution, and boiled for 60 min. The residue was separated from supernatant through filtration using a crucible and regarded as NDF. Determination of ADF was similar to that of NDF except that the solution used was an acid detergent solution. The NDF and ADF values were expressed exclusive of residual ash. For NDICP and ADICP determinations, the NDF and ADF residues were further analyzed for their CP contents, respectively [21]. Determination of these chemical constituents was performed in duplicate.

### Silage quality determination

The supernatant obtained after the ensiling procedure was determined for pH, total volatile fatty acid (VFA) concentration and ammonia concentration. Measurement of silage pH was conducted using a pH meter. Determination of total VFA concentration was according to the steam distillation method whereas ammonia was determined by employing a Conway microdiffusion method as described by Jayanegara *et al*. [22].

### *In vitro* rumen fermentation

Ground silage samples were subjected to *in vitro* incubation with rumen fluid: buffer mixture [23]. Briefly, an amount of 0.75 g of each silage sample was inserted into an incubation bottle (125 ml capacity) and then was added 75 ml rumen fluid: buffer mixture that comprised 15 ml rumen fluid and 60 ml buffer. All bottles were saturated with CO_2_ to maintain anaerobic condition before *in vitro* incubation. Bottles were closed with rubber caps and aluminum seals to start the incubation. Incubation was done in a water bath at a constant temperature of 39°C for 72 h. Gas production was vented and recorded at 2, 4, 6, 8, 10, 12, 24, 36, 48, and 72 h after incubation using a plastic syringe (equipped with a needle) with a volume of 50 ml. Manual shaking was performed to all bottles shortly after gas production measurement. Methane gas production was determined at each gas production measurement according to Fievez *et al*. [24].

Another set of bottles that received similar samples was incubated for 24 h for determination of rumen fermentation and digestibility parameters. Solid and supernatant parts in each incubation bottle were separated using a centrifuge. The resulting supernatant was subjected to analysis of total VFA and ammonia concentration as described above, similar to that of silage quality determination. The solid part was incubated again with 75 ml of pepsin-HCl 0.2 N solution for another 24 h. After the second incubation, the residue was filtered using a filter paper under vacuum. It was then dried in an oven at 105ºC for 24 h and subsequently put in a furnace at 500°C for 4 h to obtain DM and OM weight of the residue, respectively. *In vitro* DM digestibility (IVDMD) and *in vitro* organic matter digestibility (IVOMD) were calculated by the difference between initial DM and OM weight before incubation and final DM and OM weight after the two-stage *in vitro* incubation, respectively [25]. The *in vitro* incubation was performed in three replicates and allocation of samples into experimental units followed a randomized complete block design.

### Statistical analysis

Data obtained were subjected to analysis of variance with a factorial statistical model in which the first factor was different silage species (Indigofera and Moringa) and the second factor was tannin addition levels (0, 2, and 4% DM). Any significant effects of the treatments, either main factors or their interaction, were continued with a *post hoc* test, namely Duncan’s multiple range test. These statistical analyses were conducted using SAS software version 9.1. For data on chemical composition only, silage samples from three replicates were pooled into one and were analyzed in duplicate; therefore, the data were presented descriptively.

## Results

### Chemical composition and silage quality

CP contents of Indigofera and Moringa silages were comparable, i.e., slightly above 30%, expressed on a DM basis (Table-1). Contents of NDF, ADF, NDICP, and ADICP of Moringa silage were lower in comparison to Indigofera silage. Addition of tannin extract up to 4% DM did not change NDF and ADF contents of Indigofera and Moringa silages. Tannin addition at 4% increased NDICP and ADICP of Indigofera silage. A similar response was observed in Moringa silage, but it required less tannin, i.e., 2% to increase its NDICP and ADICP.

**Table-1:** Chemical composition of Indigofera and Moringa leaf silage with different level of chestnut tannin addition.

Forage	Tannin (%DM)	CP (%DM)	NDF (%DM)	ADF (%DM)	NDICP (%CP)	ADICP (%CP)
Indigofera	0	31.7	24.3	21.1	11.0	6.1
	2	31.7	23.7	21.1	11.1	6.1
	4	31.7	23.4	21.3	12.6	9.4
Moringa	0	30.2	20.7	13.6	7.4	5.4
	2	30.9	19.7	13.4	9.0	6.2
	4	30.6	19.4	13.7	9.3	6.4

ADF=Acid detergent fiber, ADICP=Acid detergent insoluble crude protein, CP=Crude protein, DM=Dry matter, NDF=Neutral detergent fiber, NDICP=Neutral detergent insoluble crude protein

Loss of DM was similar between Indigofera and moringa during ensiling either without or with tannin extract addition (Table-2). Moringa silage had lower pH than that of Indigofera silage (p<0.05), and tannin addition did not change pH of both Indigofera and moringa silages. Indigofera silage produced higher total VFA and ammonia concentrations as compared to Moringa silage (p<0.05). Higher addition level of tannin decreased total VFA and ammonia concentrations of both Indigofera and Moringa silages (p<0.05).

**Table-2:** Fermentation characteristics of Indigofera and Moringa leaf silage with different level of chestnut tannin addition.

Forage	Tannin (%DM)	DM (%)	DM loss (%)	pH	VFA (mmol/l)	NH_3_ (mmol/l)
Indigofera	0	20.1	1.56	5.37^c^	153^d^	13.0^c^
	2	20.1	2.07	5.27^bc^	143^c^	10.5^bc^
	4	20.7	2.04	5.57^c^	138^bc^	8.4^ab^
Moringa	0	20.1	1.49	5.00^ab^	138^bc^	10.9^bc^
	2	20.1	4.55	4.97^ab^	129^b^	8.4^ab^
	4	20.5	2.83	4.87^a^	116^a^	6.0^a^
SEM		0.254	0.368	0.070	3.12	0.610
p-value						
Forage		0.941	0.119	<0.001	<0.001	0.006
Tannin		0.692	0.112	0.647	<0.001	<0.001
Forage×tannin		0.989	0.287	0.178	0.545	0.976

Different superscripts within the same column are statistically different at p<0.05. DM=Dry matter, NH_3_=Ammonia, SEM=Standard error of mean, VFA=Volatile fatty acid

### *In vitro* rumen fermentation and digestibility

**Figure-1:**
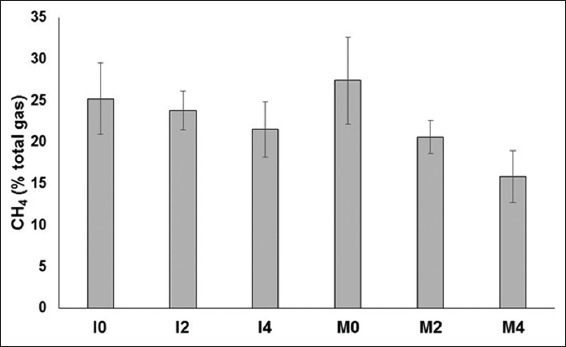
*In vitro* methane concentration (% total gas) of Indigofera and Moringa leaf silage with different level of chestnut tannin addition. I0, Indogofera leaf silage; I2, Indogofera+2% chestnut tannin; I4, Indogofera+4% chestnut tannin; M0, Moringa leaf silage; M2, Moringa+2% chestnut tannin; M4, Moringa+4% chestnut tannin. p: forage=0.282, tannin=0.027, forage×tannin=0.300. Error bar indicates standard error for each treatment.

*In vitro* gas production of Moringa silage was higher than that of Indigofera silage at 12 h incubation period (p<0.05), but it turned to be similar for 24 h and after (Table-3). Addition of tannin extract decreased *in vitro* gas production of both Indigofera and Moringa silages (p<0.05). A higher level of tannin addition reduced ruminal total VFA concentration, ammonia, IVDMD and IVOMD of Indigofera and Moringa silages (p<0.05; Table-4). The interaction between forage×tannin was significant for total VFA (p<0.05), indicating that the magnitude of decrease of the parameter was different between the two forages with increasing level of tannin addition. Addition of 2 and 4% tannin decreased ruminal total VFA concentration of Indigofera silage by 11.9 and 35.1%, respectively, whereas the decrease was 23.6 and 41.7% for Moringa silage, respectively. Tannin addition also decreased ruminal methane emission of both Indigofera and Moringa silages (p<0.05), but the emission was similar between them (Figure-1).

**Table-3:** *In vitro* gas production at a various incubation period of Indigofera and Moringa leaf silage with different level of chestnut tannin addition.

Forage	Tannin	Gas production (ml)				
	
	(%DM)	12 h	24 h	36 h	48 h	72 h
Indigofera	0	61^bc^	88^c^	98^c^	103^c^	108^c^
	2	57^b^	83^bc^	95^bc^	100^bc^	105^abc^
	4	44^a^	72^a^	89^a^	95^ab^	100a
Moringa	0	70^d^	87^c^	97^c^	101^c^	107^bc^
	2	64^cd^	82^bc^	92^abc^	99^abc^	106^abc^
	4	61^bc^	79^b^	90^ab^	94^a^	101^ab^
SEM		1.709	1.248	1.012	1.154	1.008
p-value						
Forage		<0.001	0.378	0.517	0.480	0.999
Tannin		<0.001	<0.001	0.001	0.004	0.016
Forage×tannin		0.119	0.147	0.699	0.993	0.970

Different superscripts within the same column are statistically different at p<0.05. DM=Dry matter; SEM=Standard error of mean

**Table-4:** *In vitro* rumen fermentation and digestibility of Indigofera and Moringa leaf silage with different level of chestnut tannin addition.

Forage	Tannin (%DM)	VFA (mmol/l)	NH_3_ (mmol/l)	IVDMD (%)	IVOMD (%)
Indigofera	0	134^f^	25.2^d^	70.0^c^	67.8^d^
	2	118^d^	16.9^c^	66.3^b^	64.2^bc^
	4	87^b^	15.0^ab^	62.9^a^	60.4^a^
Moringa	0	127^e^	24.7^d^	69.8^c^	66.3^cd^
	2	97^c^	15.8^bc^	68.2^bc^	64.4^bc^
	4	74^a^	13.4^a^	66.2^b^	62.5^ab^
SEM		3.82	0.832	0.519	0.545
p-value					
Forage		<0.001	0.041	0.010	0.713
Tannin		<0.001	<0.001	<0.001	<0.001
Forage×tannin		0.040	0.677	0.087	0.132

Different superscripts within the same column are statistically different at p<0.05. DM=Dry matter, IVDMD=*In vitro* dry matter digestibility, IVOMD=*In vitro* organic matter digestibility, NH_3_=Ammonia, SEM=Standard error of mean, VFA=Volatile fatty acid

## Discussion

### Chemical composition and silage quality

High CP contents observed in Indigofera and Moringa silages confirm their potency to be used as protein supplements in ruminant diets as also reported by other authors [1,2]. Tannin extract addition does not alter CP contents of the silages, but it increases NDICP and ADICP proportions to total CP. During ensiling, protein is degraded into various peptides and amino acids with lower molecular weights and more soluble [6]. The NDICP and ADICP are insoluble CP in neutral and acid detergent solutions, respectively, and tannin apparently prevents their degradation. Although tannin may also interact with fiber components, the unchanged NDF and ADF contents of the silages indicate its lower affinity as compared to tannin-protein interaction. In agreement to the present results, Adesogan and Salawu [26] observed that addition of quebracho tannin at 16 g/kg fresh weight of high pea (pea/wheat 3:1) and low pea (pea/wheat 1:3) bi-crop silages did not alter their NDF contents. Similarly, the NDF and ADF contents of perennial ryegrass silage were similar between control and the one added with mimosa tannin at 75 g/kg forage DM [27].

The generally high pH values observed in Moringa and Indigofera silages are apparently related to their high CP contents. Forages containing high CP like legumes possess higher buffering capacity as compared to low CP forages like grasses [28]. While grass silage may reach pH value around 4.0 or less [27,29], it is normally hard for legume silage to obtain a pH value of 4.5 or less [30]. Lower pH found in Moringa silage as compared to that of Indigofera is apparently related to its lower NDF and ADF contents. This indicates that Moringa silage contains more readily available and fermentable carbohydrate to result in more lactic acid concentration that contributes to lower pH value. It has been widely known that a primary factor determining silage quality is the concentration of readily available or water-soluble carbohydrate (WSC). Lactic acid bacteria proliferate and utilize WSC to produce lactic acid [31]. As the lactic acid is produced and increases in concentration, it decreases pH of the silage below a certain critical point to prevent the growth of undesired or spoilage microbes [32].

Higher ammonia concentration of Indigofera silage as compared to that of Moringa silage is apparently related to its slightly higher CP content. A lower concentration of ammonia by addition of tannin is possible since tannin protects plant protein from microbial degradation and fermentation during ensiling. Free phenolic groups present in tannin enable the compound to form tannin-protein complexes [33]. Tannin may also bind to enzyme released by proteolytic microbes in silage and therefore reduce their activity [26]. In agreement with the present finding, Ding *et al*. [34] observed that addition of tannic acid, a simple form of tannin, decreased ammonia nitrogen, non-protein nitrogen, and amino acid concentrations in alfalfa silage.

Further, in a recent meta-analysis study, it was revealed that addition of tannin extracts (from various sources) lowered concentrations of butyric acid, soluble nitrogen, non-protein nitrogen and ammonia nitrogen of silages, and tended to reduce free amino acid concentration [35]. From these facts, it is apparently evident that addition of tannin to high protein silages may lower the extent and rate of proteolysis during ensiling and therefore improve their quality.

### *In vitro* rumen fermentation and digestibility

Higher *in vitro* gas production of moringa silage than that of Indigofera during early incubation hours seems to be caused by its higher fermentable carbohydrate. This is indicated by the lower NDF and ADF contents of Moringa silage as compared to Indigofera silage. Gas in the *in vitro* rumen incubation system is produced by nutrient degradation and fermentation, particularly carbohydrate. Although protein fermentation also contributes to gas production, it stoichiometrically produces considerable gas in comparison to carbohydrate, whereas the contribution of fat to gas production is negligible [36]. Therefore, extent and rate of gas production are primarily determined by content and type of carbohydrate present in the substrate. Accordingly, Krieg *et al*. [37] investigated *in vitro* ruminal fermentation of grains from different rye, triticale, and barley genotypes. The authors observed that, supporting the current study, starch concentrations in these grains were positively correlated with gas production measured at 24 h after incubation, potential gas production, and gas production rate. Conversely, their NDF and ADF concentrations were negatively correlated with potential gas production and gas production rate.

Lower *in vitro* gas production, ruminal total VFA, ammonia concentration, IVDMD, and IVOMD of both Indigofera and Moringa silages at the increasing level of tannin addition indicate that the compound reduces rumen fermentation. Such responses are possible due to the interaction between tannin with macromolecules particularly protein and carbohydrate as described above, thus protecting their degradation and fermentation in the rumen. VFA and ammonia in the rumen are fermentation products of mainly carbohydrate and protein, respectively [38,39]. Confirming the results obtained, a number of studies have reported negative correlations between tannin and total VFA, ammonia concentration, or *in vitro* digestibility [40-42]. Despite such facts, the presence of tannin under ruminal environment does not always mean negative with regard to its effect on animal performance. Tannin protects protein in the rumen [10], increases proportion of rumen undegradable protein [43], increases supply of metabolizable protein for the host animal [11], and enhances animal production performance [12,44].

Although tannin is added for conserving protein and thus improving silage quality, its effect on mitigating ruminal methane emission is obvious. Methane mitigating effect of tannin is possible since tannin has been shown to decrease the methanogen population, the microbial group responsible for methanogenesis. Jayanegara *et al*. [45] observed that addition of purified tannins from chestnut, sumach, mimosa, or qu bracho at 1 mg/ml addition level decreased methanogen population in the rumen *in vitro*. The methanogen population after such addition became 63.3–77.7% from the population in the control treatment (without purified tannin addition). Further, tannin may also reduce protozoa population whereby part of the methanogen lives in symbiosis with the fauna. Bhatta *et al*. [14] reported that increasing tannin concentration from 0% to 25% of total mixed ration (DM basis) linearly decreased protozoa population. Apart from its antimicrobial activity on methanogen and protozoa in the rumen, tannin decreases hydrogen production, the main substrate for methanogenesis through the reduction in nutrient degradation particularly fiber. This was indicated by the lower IVDMD, and IVOMD observed in the present study. It had been demonstrated that incubation of *Lotus pedunculatus* (containing condensed tannin) produced hydrogen by approximately 50% as compared to that of *Medicago sativa* (no tannin) [46]. Tannin may also serve as a hydrogen sink that competes with methane formation [47].

## Conclusion

Tannin extract can reduce proteolysis of high protein silage from Moringa and Indigofera leaves. Therefore, the natural compound is promising to be used as a silage additive particularly when the ensiled material is rich in protein. Tannin retains its biological activity during ensiling and thus may provide additional benefits when being used in silage, i.e., as a protein by-pass and methane-mitigating agents. Further, *in vivo* experiments are required to observe the effects of tannin addition in silage on animal production performance.

## Authors’ Contributions

AJ designed and supervised the experiment, analyzed the data, drafted, and revised the manuscript. AY performed the experiment and collected the data. LK supervised the experiment and revised the manuscript. All authors read and approved the final manuscript.
